# Complete Genome Sequence of *Mycobacterium tuberculosis* Clinical Isolate Spoligotype SIT745/EAI1-MYS

**DOI:** 10.1128/genomeA.00323-16

**Published:** 2016-05-19

**Authors:** S. Suraiya, N. Semail, M. F. Ismail, J. M. Abdullah

**Affiliations:** a Medical Microbiology Department, School of Medical Sciences, Universiti Sains Malaysia, Kubang Kerian, Kelantan, Malaysia; b Center for Neuroscience Services and Research (P3Neuro) Health Campus, Universiti Sains Malaysia, Kubang Kerian, Kelantan, Malaysia

## Abstract

*Mycobacterium tuberculosis* is known to cause pulmonary and extrapulmonary tuberculosis. This organism showed special phylogeographical specificity. Here, we report the complete genome sequence of *M. tuberculosis* clinical isolate spoligotype SIT745/EAI1-MYS, which was isolated from a Malaysian tuberculosis patient.

## GENOME ANNOUNCEMENT

Tuberculosis (TB) remains a major global public health problem. TB is an ancient infectious disease caused by *Mycobacterium tuberculosis*. It ranks as the second leading cause of death from an infectious disease worldwide after human immunodeficiency virus (HIV) (1).

Recently, spoligotyping of *M. tuberculosis* isolated in Malaysia by our research group (2) highlighted that the East-African-Indian (EAI) lineage is the most prevalent lineage in Malaysia. Deeper analysis revealed that there is a phylogeographical specificity of SIT745/EAI1-MYS for Malaysia, and there is probable ongoing evolution with locally evolved strains sharing a specific signature characterized by the absence of spacers 37, 38, and 40 in the spoligotyping results.

*M. tuberculosis* strain SIT745/EAI1-MYS was isolated from a tuberculosis patient who presented to Hospital Universiti Sains Malaysia (HUSM), a tertiary teaching hospital located in the northeastern region of Malaysia.

Genomic DNA was extracted from a 3-week-old culture by a genomic DNA extraction kit (Qiagen, Hilden, Germany). The purified genomic DNA was subjected to whole-genome shotgun sequencing on an Illumina MiSeq (Illumina, Inc., USA) platform with 2 × 101-bp read length. The raw reads were trimmed using Trimmomatic (3) and Scythe (https://github.com/vsbuffalo/scythe). SGA (4) was used for error correction. The trimmed reads were subjected to *de novo* assembly with IDBA-UD 1.0.9 (5). Gene prediction was performed with the prokaryotic gene prediction algorithm Prodigal (version 2.60) (6), while rRNAs were predicted with RNAmmer (7). tRNA prediction was performed using Aragorn (8). Subsequently, the genome sequence was annotated with BLASTn (9, 10) against the Swiss-Prot database.

The *M. tuberculosis* SIT745/EAI1-MYS genome, consisting of 4,371,919 bases, was obtained at 194× coverage, and its G+C content is 65.5%. The total number of protein-coding genes is 4,105.

The sequenced genome of *M. tuberculosis* SIT745/EAI1-MYS serves as a foundation through which we can better understand the *M. tuberculosis* species that has a specific occurrence in Malaysia.

### Nucleotide sequence accession numbers.

This whole-genome shotgun project has been deposited at DDBJ/ENA/GenBank under the accession no. LUDZ00000000. The version described in this paper is version LUDZ01000000.
